# Prediction of early urinary continence after radical prostatectomy based on preoperative pelvic floor parameters: a retrospective study

**DOI:** 10.3389/fonc.2025.1591435

**Published:** 2025-12-10

**Authors:** Shuhui Yu, Jianing Han, Yanbo Huang, Ziwei Liu, Kaiyue Chen, Kai Zhang, Yisen Meng, Xinyan Che

**Affiliations:** Peking University First Hospital Department of Urology, Beijing, China

**Keywords:** urinary incontinence, prostate cancer, radical prostatectomy, pelvic floor muscle parameters, prediction

## Abstract

**Background:**

Postoperative urinary incontinence is one of the most significant complications following radical prostate cancer surgery. We aimed to predict the risk of urinary incontinence within one month of radical prostatectomy (RP) using preoperative physiological parameters of the pelvic floor muscles.

**Methods:**

This is a retrospective study with a convenience sample. A cohort of 188 patients with prostate cancer was recruited from March to December 2023 from a single urology department at Peking University First Hospital. The cohort was divided into a training set of 132 patients and a validation set of 56 patients at a 7:3 ratio. This study used multivariate logistic regression analysis to predict urinary incontinence and calculated the area under the receiver operating characteristic curve (AUC) for model validation. Nomograms and calibration plots were generated for training sets. Preoperative and operative parameters were collected, including age, body mass index (BMI), International Prostate Symptom Score (IPSS), prostate-specific antigen (PSA) level, Gleason score, surgical method, urethral reconstruction, lymph node dissection, nerve-sparing status, catheterization duration, D’Amico risk classification, American Society of Anesthesiologists (ASA) score, Charlson Comorbidity Index, postoperative duration, prostate volume, and pelvic floor muscle parameters.

**Results:**

The incidence of urinary incontinence within a month after RP was 78.7%. Advanced age and low fast-twitch muscle scores have emerged as independent risk factors for urinary incontinence. Patients older than 70 had a 6.283-fold higher risk of incontinence compared to those younger than 60 (95% CI: 1.47-26.95). The fast-twitch muscle score was significantly associated with the risk of incontinence (OR = 1.25; 95% CI: 1.05-1.49). The AUC was 0.764 (95% CI: 0.675-0.854) for the training set and 0.776 (95% CI: 0.644-0.908) for the validation set, with calibration plots indicating high model accuracy.

**Conclusions:**

Advanced age and low fast-twitch muscle scores in functional level were significant risk factors for RP. This risk predictive model enables healthcare professionals to perform accurate preoperative risk assessments and predictions based on patients’ individualized indicators, and provide tailored postoperative rehabilitation strategies.

## 1 Introduction

Prostate cancer is a common malignant tumour that affects the genitourinary system in elderly men. Its incidence and mortality rates are ranked second and fifth, among male malignant tumours worldwide, and sixth and seventh, respectively, among Chinese males (1). Radical prostatectomy (RP) is a common treatment for localised prostate cancer. However, postoperative urinary incontinence is a significant complication second only to erectile dysfunction (2). Urinary continence was defined as the usage of 0 pads per day. According to a longitudinal study of 1,042 patients with RP, 61.4%, 39.5%, and 41.9% of patients experienced urinary incontinence at 6, 12, and 24 months postoperatively, respectively (3). Additionally, 25.2%, 14.3%, and 8.7% of the patients rated urinary incontinence as a moderate-to-major problem in the same intervals. This condition significantly affects patients’ quality of life and overall satisfaction (4).

Numerous studies have shown that advanced age, large prostate volume, severe lower urinary tract symptoms, a high comorbidity index, and short membranous urethral length (MUL) are significant risk factors for post-RP urinary incontinence (5, 6). The extent of nerve sparing during surgery also affects the likelihood of postoperative urinary incontinence (5), whereas other surgical factors are yet to be definitively confirmed (6). According to the 2019 American Urological Association and Society of Urodynamics, Female Pelvic Medicine & Urogenital Reconstruction (AUA/SUFU) clinical guidelines, engaging in pelvic floor muscle exercises both before and immediately after surgery has shown considerable benefits for patients in terms of aiding their recovery from urinary incontinence (7). A retrospective study previously investigated the impact of preoperative pelvic floor electromyography parameters on the risk of urinary incontinence following robot-assisted radical prostatectomy (8). However, this earlier investigation fell short of predictive measurement efficiency. In light of this, the current study aimed to explore the effect of preoperative pelvic floor electromyography parameters on the risk of urinary incontinence at one month post-RP.

The objective of this study was to construct and internally validate predictive models and utilise ROC curves to facilitate the personalised pelvic floor muscle exercise regimen. Finally, the implementation of nomograms will be employed for data presentation. This approach is expected to assist patients in accurately predicting the risk of developing urinary incontinence, thereby enabling more targeted and effective preoperative and postoperative care strategies.

## 2 Materials and methods

### 2.1 Design

A retrospective cohort study was conducted with a convenience sample by continuous recruitment from a single urology department in Peking University First Hospital.

### 2.2 Setting and participants

We included 188 patients of RP who were treated between March and December 2023. The inclusion criteria of eligible patients were: (i) Diagnosed with prostate cancer through pathological results, (ii) Planning to undergo robot-assisted laparoscopic radical prostatectomy (RARP) or laparoscopic radical prostatectomy (LSRP) at a scheduled time, (iii) Age ≥ 40 years, (iv)Patients who received neoadjuvant endocrine therapy (ADT) before surgery also eligible. The exclusion criteria were as follows: (i) History of confirmed diagnosis of urinary incontinence, (ii) Preoperative radiotherapy or chemotherapy, (iii) Standard range of resection not performed during surgery for various reasons or incomplete surgical records, (iv) Lost to follow-up or incomplete follow-up, and (vi) Diseases affecting bladder function, such as neurogenic bladder.

### 2.3 Instruments and measures

#### 2.3.1 Demographic characteristics and clinical data

A single surgeon performed all the surgeries. Two uniformly trained urology nurses collected baseline data from the patients before surgery through questionnaires and a medical record review. We included the following data: patient demographics, such as age, body mass index (BMI), preoperative International Prostate Symptom Score (IPSS), preoperative Prostate-Specific Antigen (PSA) level, and prostate volume (results of urological ultrasound all from our center). Additional variables considered were the Gleason score, T staging, surgical type, urinary tract reconstruction, lymph node dissection, nerve-sparing status (partial or none), duration of catheterisation, and postoperative duration.

Patients were categorised using several classifications. First, the D’Amico risk classification divides patients into low-, medium-, and high-risk groups based on PSA levels, tumour grade (Gleason score), and clinical stage (T stage). Secondly, the American Society of Anaesthesiologists (ASA) physical status classification assigns grades ranging from I to VI. Third, the Charlson Comorbidity Index (CCI) was used to quantify comorbidities (9); a CCI score of 4 or higher indicated a high-risk group, whereas a score below 4 indicated a low-risk group, predicting postoperative complications. Lastly, urinary incontinence was evaluated based on pad usage per day. Patients who used one or fewer pads per day were considered to have achieved urinary control; otherwise, they were considered to have urinary incontinence. The rate of urinary incontinence was based on data collected one month after RP surgery.

#### 2.3.2 Pelvic floor assessment

The assessment includes five parameters: (1) Pre-resting phase electromyography (EMG) average value: A test value greater than 4 µV indicates excessive pelvic floor muscle tension; (2) Fast-twitch muscle phase (Type II fibres) peak value with a test value less than 70 µV indicating insufficient fast-twitch muscle strength; (3) Slow-twitch muscle phase (Type I fibres) average value with a test value less than 50 µV indicating insufficient slow-twitch muscle strength; (4) Endurance test phase average value with a test value less than 40 µV indicates decreased slow-twitch muscle endurance and fatigue resistance; (5) Post-resting phase EMG average value with a test value greater than 4 µV indicates high muscle tone in the pelvic floor during the resting state after exercise.

### 2.4 Data collection

All patients scheduled for RARP or LSRP underwent a preoperative assessment conducted by a specialised nurse and biofeedback therapist who was fully responsible for the assessment of patients (standardized procedural controls), exercise plans, and effect evaluation in this project. This evaluation utilises a biofeedback system to measure pelvic floor EMG parameters, specifically employing the Glazer assessment. The equipment used was a Mailande Bioelectric Stimulator (urology version), Model MLD-B2PLUS, with system version MNNK (BS-STD) V6.8.13.16-(CS)V2.00-(FW) V3.0.

The assessment procedure involved the patient lying in the supine position with the head slightly elevated. The operator inserts a rectal electrode into the patient’s anus and places abdominal electrode pads on the rectus abdominis muscle. Surface EMG signals were collected from the pelvic floor using a rectal electrode. The patient followed voice and visual prompts to perform a series of pelvic floor muscle contractions and relaxations. During contraction, the abdominal, gluteal, and thigh adductor muscles remained as relaxed as possible.

### 2.5 Statistical analysis

In this study, normally distributed measurement data are expressed as mean ± SD, while skewed distribution measurement data are represented as [M (P25, P75)]. Categorical data are presented as n (%). The data were randomly divided into a training set (70%) and a validation set (30%) in a 7:3 ratio. Baseline information for the two groups of patients was compared using the training set data, employing univariate and multivariate binary logistic stepwise regression analysis to explore independent risk factors for urinary incontinence (with univariate analysis p<0.1 entering the multivariate model). Risk factors for urinary incontinence identified from univariate analysis and clinically significant variables were incorporated into the predictive model to identify independent risk factors affecting early postoperative urinary incontinence, with p<0.05 considered statistically significant. To reduce high collinearity among the predictors in the model, collinearity diagnostics were used, and variables with a variance inflation factor (VIF) exceeding 10 were excluded. All the included variables had missing values (< 20%). The discriminative power of pelvic floor muscle assessment results for RP urinary incontinence was evaluated by calculating the area under the receiver operating characteristic (ROC) curve (AUC). The model results were visualised using a nomogram, and internal validation of the training set was performed using the bootstrap method with 1000 iterations. To validate the effectiveness of the model, the AUC under the ROC curve (AUC) was calculated using the validation dataset to assess the predictive power for urinary incontinence. All analyses were conducted using R3.6.0 (R Foundation for Statistical Computing, Vienna, Austria, 2019).

## 3 Results

### 3.1 Occurrence and characteristics of RP

One month after RP, 188 patients were included in the study. The demographic characteristics of the participants are shown in **Table 1**, with a urinary incontinence rate of 78.7%. Nerve-sparing procedures were performed in 16 (8.5%), lymph node dissection in 44 (23.4%), and urethral reconstruction in 153 (81.4%) patients. Eleven (5.9%) patients underwent LSRP, and 177 (94.1%) underwent RARP. Based on the D’Amico risk classification, 146 (77.7%) patients were high-risk, 32 (17.0%) were intermediate-risk, and 10 (5.3%) were low-risk. Four patients had positive lymph nodes. All patients had normal muscle tone at rest post-procedure, with only two (1.1%) showing muscle tension at rest. The median maximum fast muscle score was 2.64 (1.57, 5.21) and the median maximum slow muscle score was 2.04 (1.39, 3.91). None of the patients met the scoring criteria for fast muscle performance and only one patient met the criteria for slow muscle performance (dichotomized “meeting performance threshold”).

**Table 1:** Demographic and clinical data of patients.

Variable	Training cohort		Texting cohort	
	Total(132)	Missing values, n(%)	Total(56)	Missing values, n(%)
Age/years, n (%)		0		0
≤60	34(25.8)		12(21.4)	
60-70	72(54.5)		30(53.6)	
≥70	26(19.7)		14(25.0)	
Pelvic floor muscle assessment		0		0
Pre-resting Assessment, n (%)				
Normal muscle tension	130(98.5)		56(100)	
High muscle tension	2(1.5)		0	
Fast muscle score, M(P_25_, P_75_)	2.7(1.6, 5.3)	0	2.5(1.4, 4.2)	0
Slow muscle score, score, M(P_25_, P_75_)	2.1(1.4, 4.0)	0	1.9(1.3, 3.5)	0
Endurance assessment, M(P25, P75)	2.1(1.2, 3.9)	0	2.1(1.3, 3.7)	0
BMI/(kg/m^2^),n (%)		0		0
BMI<24	55(41.7)		24(42.9)	
24≤BMI<28	56(42.4)		26(46.4)	
BMI≥28	21(15.9)		6(10.7)	
Surgical method, n (%)		0		0
LSRP	8(6.1)		3(5.4)	
RARP	124(93.9)		53(94.6)	
IPSS score, M(P_25_, P_75_)	6.0(3.0, 12.0)	0	6.5(2.0, 15.8)	0
ASA, n (%)		0		0
I or II	100(75.8)		39(69.6)	
III	32(24.2)		17(30.4)	
CCI		0		0
0	72(54.5)		33(58.9)	
1 and 2	57(43.2)		22(39.3)	
3	3(2.3)		1(1.8)	
Biopsy Gleason score		0		0
6	13(9.8)		5(8.9)	
7	85(64.4)		36(64.3)	
8	11(8.3)		5(8.9)	
9-10	23(17.4)		10(17.9)	
Clinical T stage		0		0
2	51(38.6)		26(46.4)	
3	58(43.9)		22(39.3)	
Neoadjuvant endocrine therapy	23(17.4)		8(14.3)	
Lymph node				
Negative	129(97.7)	0	55(82.1)	0
Positive	3(2.3)		1(17.9)	
D’Amico risk Classification, n (%)		0		0
Low	8(6.1)		2(3.6)	
Medium risk	22(16.7)		10(17.9)	
High risk	102(77.3)		44(78.6)	
Preservation of nerves,n (%)		0		0
Fully or partially	10(7.6)		6(10.7)	
No	122(92.4)		50(89.3)	
Lymphatic drainage		0		0
Yes	33(25.0)		11(19.6)	
No	99(75.0)		45(80.4)	
Urinary reconstruction, n (%)		0		0
Yes	105(79.5)		48(85.7)	
No	27(20.5)		8(14.3)	
Length of surgery(LOS)/min, n(%)		0		0
LOS ≤ 120	48(36.4)		8(14.3)	
120<LOS<180	66(50.0)		29(51.8)	
LOS≥180	18(13.6)		19(33.9)	
Prostate volume/ml,(mean ± SD)	41.1 ± 21.2	19(14.4)	46.1 ± 25.5	12(21.4)
Indwelling catheterization/week,(mean ± SD)	2.0 ± 0.1	0	2.04 ± 0.2	0

### 3.2 Predicting the incontinence of RP with one month after surgery

The dataset was split in a 7:3 ratio, resulting in a training set of 132 patients and a validation set of 56 patients. In the training set, the univariate logistic regression analysis identified the following variables: pre-rest, fast muscle, slow muscle, endurance, post-rest, and age (**Table 2**). Multivariate logistic regression analysis revealed that only age and fasting muscle scores were independent predictors of post-RP urinary incontinence (**Table 3**). Specifically, patients aged 60 years or younger were 6.283 times more likely to achieve urinary continence compared to patients older than those aged > 70 years (95% CI, 1.47-26.95; P = 0.013). The fast muscle score was closely associated with recovery from urinary incontinence (OR = 1.25; 95% CI, 1.05-1.49; P = 0.014).

**Table 2:** Univariate Logistic regression analysis of the training set.

Variable	OR(95%CI)	P
Age/years, n (%)		
≤60	4.25(1.25-15.51)	0.021
60-70	1.66(0.55-4.98)	0.368
≥70	ref.	
Pelvic floor muscle assessment		
Fast muscle score, M(P25, P75)	1.26(1.09-1.45)	0.002
Slow muscle score, score, M(P25, P75)	1.29(1.06-1.57)	0.013
Endurance assessment, M(P25, P75)	1.13(0.98-1.31)	0.097
BMI/(kg/m2),n (%)		
BMI<24	ref.	
24≤BMI<28	0.78(0.30-1.74)	0.474
BMI≥28	0.83(0.26-2.68)	0.759
Surgical method, n (%)		
LSRP	ref.	
RARP	2.33(0.28-19.72)	0.437
IPSS score, M(P25, P75)	1.02(0.97-1.07)	0.501
ASA, n (%)		
I or II	ref.	
III	1.06(0.42-2.66)	0.909
CCI		
0	ref.	
1 and 2	1.48(0.66-3.34)	0.341
3	1.90(0.16-22.40)	0.610
Biopsy Gleason score		
6	ref.	
7	0.83(0.21-3.36)	0.798
8	2.78(0.48-16.03)	0.253
9-10	1.46(0.31-6.98)	0.637
Clinical T stage		
2	ref.	
3	1.78(0.71-4.47)	0.221
Neoadjuvant endocrine therapy	2.04(0.65-6.41)	0.221
D’Amico risk Classification, n (%)		
Low risk	ref.	
Medium risk	0.78(0.14-4.21)	0.771
High risk	0.46(0.10-2.07)	0.310
Preservation of nerves,n (%)		
Fully or partially	ref.	
No	0.73(0.18-3.00)	0.660
Lymphatic drainage		
Yes	0.80(0.31-2.06)	0.639
No	ref.	
Urinary reconstruction, n (%)		
Yes	0.89(0.34-2.36)	0.819
No	ref.	
Length of surgery(LOS)/min, n(%)		
LOS ≤ 120	0.77(0.23-2.65)	0.682
120<LOS<180	0.83(0.26-2.69)	0.759
LOS≥180	ref.	
Prostate volume/ml,(mean ± SD)	1.01(0.99-1.03)	0.599

**Table 3:** Final predictive model for urinary continence after radical prostatectomy in training cohort.

Predictor	β coefficient	OR(95%CI)	P-value
Fast muscle score	0.221	1.25(1.05-1.49)	0.014
Slow muscle score	0.239	1.27(0.90-1.79)	0.174
Endurance assessment	-0.157	0.86(0.62-1.17)	0.332
Age/years, n (%)			
60-70	1.031	2.81(0.71-11.08)	0.141
≤60	1.838	6.28(1.47-26.95)	0.013

**Figure 1:**
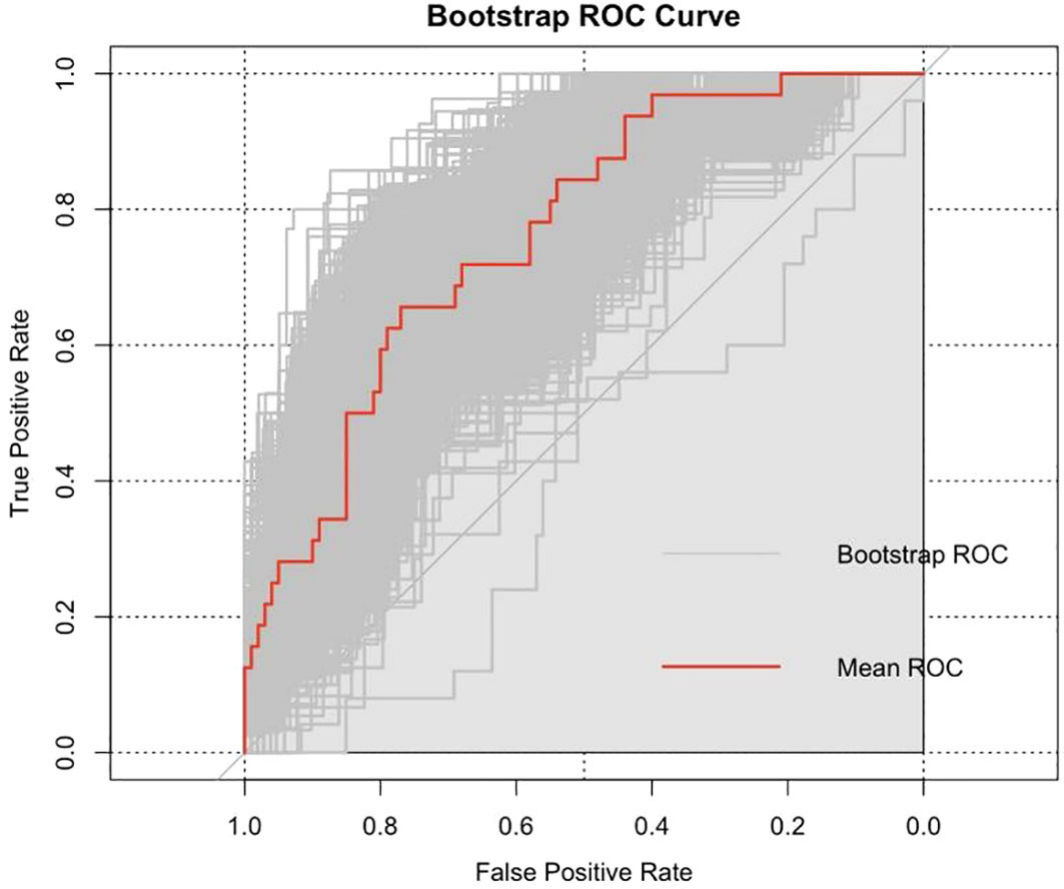
The AUC of the training cohort.

**Figure 2:**
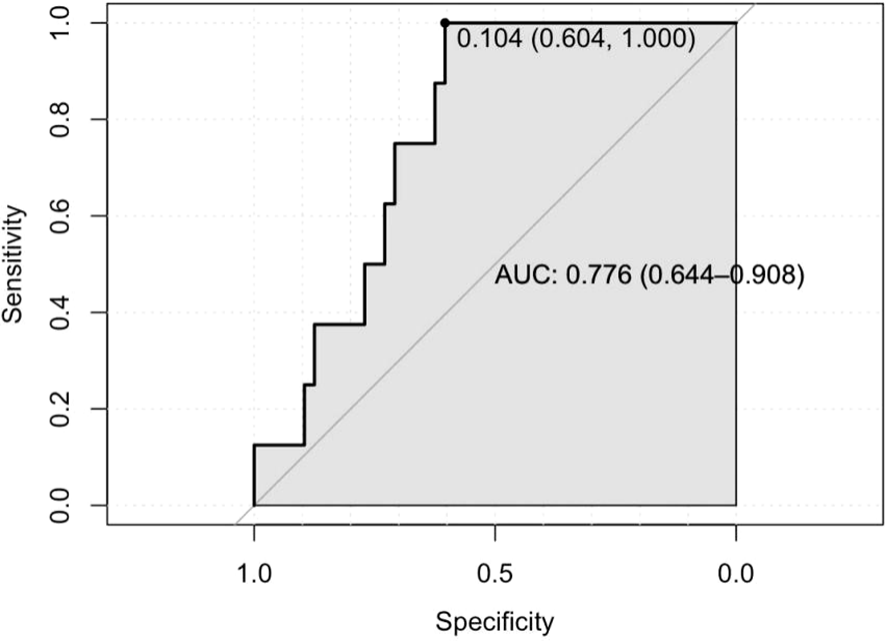
The AUC of the texting cohort.

**Figure 3:**
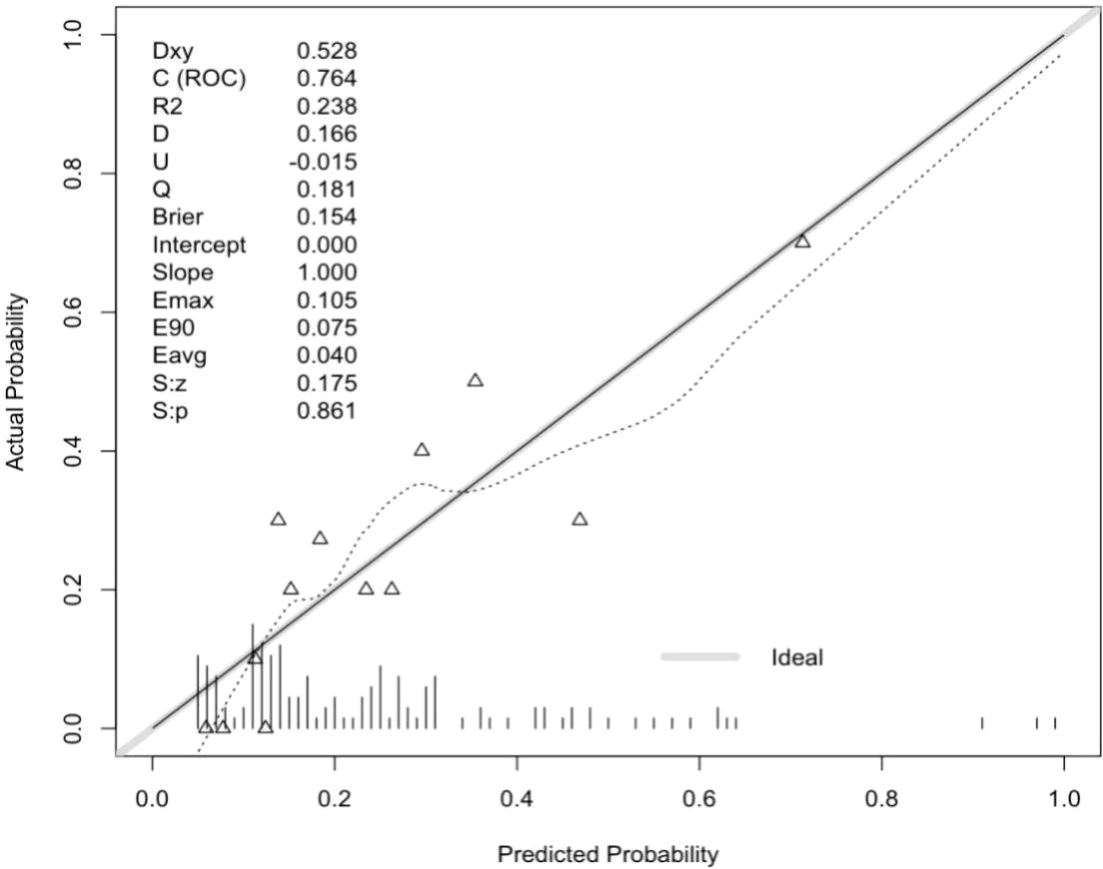
Calibration plots for training cohort data.

**Figure 4:**
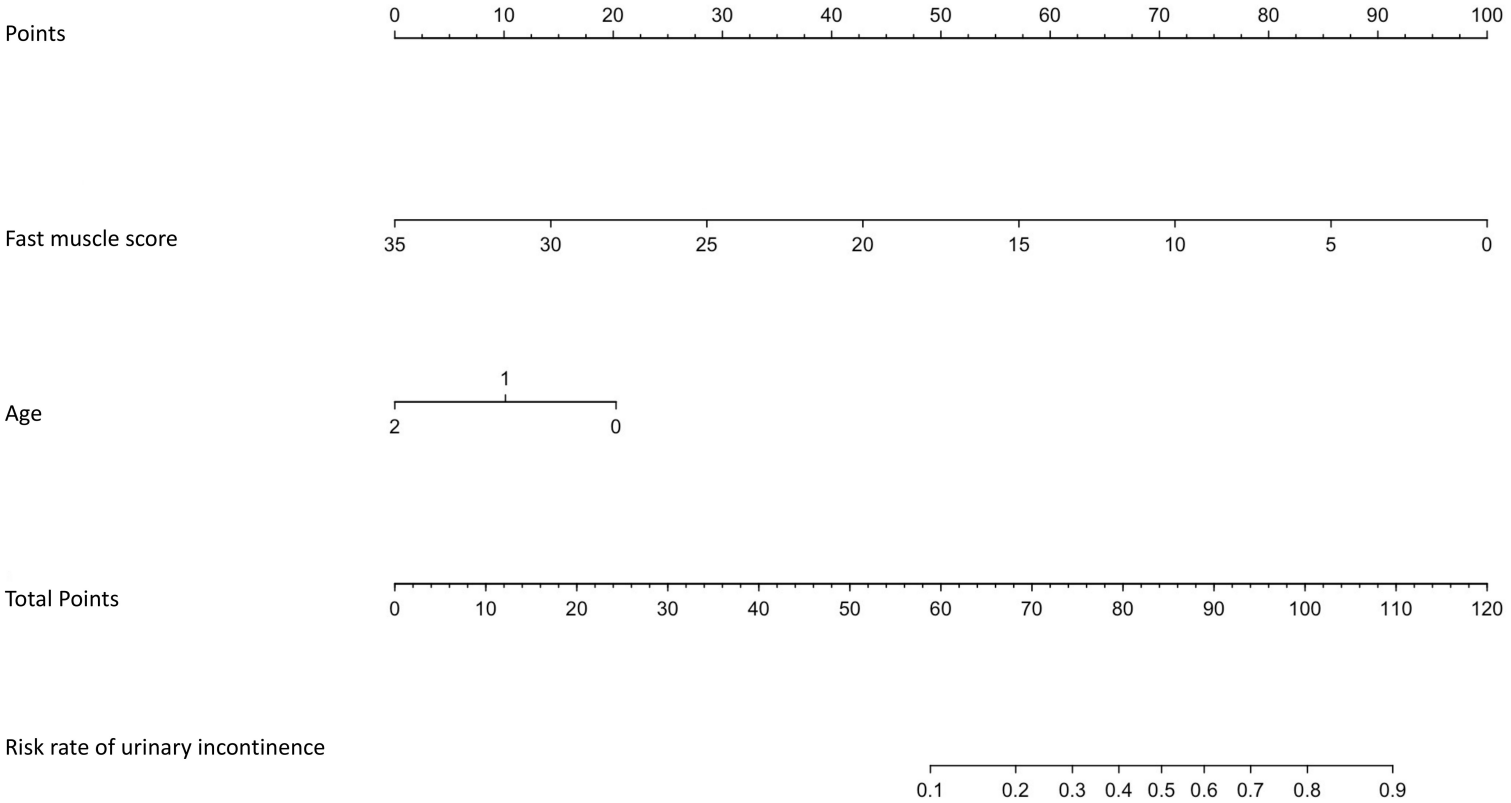
The nomogram of the training cohort data. Footnotes: 2 indicates under 60 years old, 1 indicates 60 to 70 years old, and 0 indicates over 70 years old. Peak value with a test value in the fast-twitch muscle phase. An example of calculating the incidence of postoperative urinary incontinence after RP based on age and fast-twitch muscle score. To use the nomogram, a clinician would first identify the patient’s age group and preoperative fast-twitch muscle score on their respective axes. For example, a patient older than 70 years of age (corresponding to 20 points on the ‘Points’ scale) with a fast-twitch muscle score of 15 µV (corresponding to 55 points) would accumulate a total of 75 points. By drawing a vertical line down from ‘68’ on the ‘Total Points’ axis to the final axis, the clinician can estimate the patient’s risk of urinary incontinence to be approximately 35%. This intuitive tool can facilitate personalized patient counseling and help in managing postoperative expectations.

The Hosmer-Lemeshow goodness of fit test for both the training (X^2^ = 12.516, P = 0.130) and validation sets (X^2^ = 10.883; P = 0.208) showed P≥0.05, indicating good model fit. The area under the curve (AUC) in **Figure 1** for the training set was 0.764 (95% CI: 0.675–0.854) and that for the validation set was 0.776 (95% CI: 0.644–0.908) in **Figure 2**, demonstrating that the discriminative ability of the model was acceptable. The calibration plot showed an overall good or bad agreement between the predicted and observed RP patients according to the training set, as shown in **Figure 3**. Based on the results of the multivariate logistic regression analysis, we plotted the nomogram shown in **Figure 4**.

## 4 Discussion

This study validated that age and the fast-twitch muscle score had significant predictive value for urinary continence at 1 month after surgery. Building on existing evidence that MUL is an important anatomical predictor of postoperative continence, our findings complement current understanding from a functional perspective (10).

This study found that advanced age was an independent risk factor for urinary incontinence after RP and could be used for the early identification of such risks. Multiple studies (4, 6) have shown that older age leads to more challenging recovery from postoperative urinary incontinence and serves as a significant predictor of recovery outcomes following RP. Research by Novara (11) reported that younger patients had significantly better urinary continence within one year postoperatively than older patients. Matsushita (12) conducted a study involving 2,849 patients with RP and concluded that advanced age was an independent predictor of urinary incontinence at both 6 and 12 months post-surgery. This issue arises mainly because the striated muscle cells of the external urethral sphincter undergo apoptosis with ageing (13), leading to a decline in related functions and a reduction in both the number and concentration of muscle cells in the urethral sphincter (6). Additionally, elderly patients often have enlarged prostates, which alter bladder and urethral function, resulting in a higher incidence of lower urinary tract symptoms than in younger patients. These findings highlight the need for healthcare professionals to inform elderly patients scheduled for RP about the risks of postoperative urinary incontinence and implement more targeted interventions. For example, proactively promoting pelvic floor muscle exercises tailored for older patients can help shorten the recovery period for postoperative urinary incontinence and improve patients’ ability to independently control urination.

In this study, the AUC of the ROC curve for the training set was 0.764 and that for the validation set was 0.776. These showed both models generalized well, had reasonable sampling, and carried a low risk of overfitting.Our results were higher than those reported by Jeong et al. (14). This retrospective cohort study included 1,210 RP (RARP and open RP) patients and considered variables such as age, BMI, PSA levels, prostate volume, MUL, Charlson comorbidity score, surgery type (open RP vs. robot-assisted RP), nerve-sparing status, surgery duration, pathological Gleason score, and pathological T staging. The study identified the risk factors for urinary incontinence one month post-RP, including age, MUL, and surgery type, with an AUC of 0.641. However, our findings were lower than those of another retrospective study (15) that included 154 patients with LSRP and reported an AUC of 0.914. They evaluated independent predictors of immediate urinary control within seven days of catheter removal without using pads, including age, BMI, and pelvic floor anatomical parameters (such as MUL). Jeong et al. (14) also reported predictors for urinary incontinence at three months post-RP, including age, MUL, and surgery type, with an AUC of 0.672. The predictors at 12 months post-RP included age, MUL, surgery type, nerve-sparing status, and prostate volume, with an AUC of 0.725. These studies indicated differences in the predictors of post-RP urinary incontinence based on follow-up time and the included variables. Therefore, the fast-twitch muscle score remains a potential functional predictor.

In this study, the fast-twitch muscle score was considered a predictor of urinary incontinence following RP. The objective for this high-risk group is not necessarily to restore muscle function to the level of a younger person, but rather to improve their baseline strength and coordination to a point that is functionally sufficient for continence. Even a modest improvement can translate into a significant reduction in leakage episodes, decreased pad usage, and a better quality of life. A primary goal of PFMT is not just to build muscle mass, but to improve neuromuscular control. The training helps patients learn to consciously, correctly, and most importantly, quickly contract their pelvic floor muscles in anticipation of or during moments of increased intra-abdominal pressure (e.g., coughing, sneezing, or rising from a chair). This improved mind-muscle connection is a learned skill that can be developed even when baseline muscle strength is low. However, some studies (8) suggest that preoperative pelvic floor endurance is a predictor of urinary incontinence three months post-RP, showing differing results. The theory behind pelvic floor muscle training (PFMT) is to contract the pelvic floor muscles selectively and repeatedly, thereby increasing muscle mass, strength, and endurance to compensate for smooth and striated muscle loss during RP. Moreover, preoperative PFMT enables patients to learn the necessary exercises, allowing them to begin training immediately after catheter removal (16). However, the mechanism through which PFMT improves postoperative urinary men remains unclear. This may enhance the sphincter mechanism by strengthening striated muscle function and/or enhancing the support system of the levator ani muscle (17). PFMT acts as a form of strength training that can induce hypertrophy and increase the force-generating capacity of these remaining muscle cells. In clinical practice, men may experience urinary incontinence or “leakage” while lying down, standing up, or performing activities like coughing, standing, lifting heavy objects, and walking after RP. Stress urinary incontinence (SUI) is the most common manifestation post-RP, triggered by activities such as sneezing, coughing, bending, lifting, and changes in body position (18). A prospective study reported that pelvic floor muscle strength impacts urinary incontinence occurrence more than endurance (19). PFMT significantly boosts strength and endurance, with the changes most noticeable between the third and sixth months postoperatively, with endurance gradually improving. The first month post-surgery sees the most significant improvement in incontinence (reduced leakage volume). There is a functional relationship between strength and endurance: as strength increases, so does endurance. Compared to endurance, pelvic floor muscle strength has a greater impact on incontinence, with higher preoperative muscle strength correlating with lower incontinence rates. PFMT typically emphasise concentric contractions of the pelvic floor muscles in static positions like sitting, rather than during dynamic functional tasks, such as standing, walking, or lifting. However, these activities often trigger symptoms of post-RP incontinence symptoms. Thus, this instructional approach may result in individuals not performing these exercises correctly, thereby failing to achieve the intended pelvic floor muscle adaptation. Milios et al. (20) suggest that preoperative PFMT significantly affects post-RP incontinence. The intervention group started PFMT five weeks preoperatively and continued post-catheter removal for up to three months postoperatively. The control group performed three sets of ten contractions daily in the supine, sitting, and standing positions, while the intervention group performed rapid and slow contractions, totalling 120 contractions daily. Results showed that patients undergoing more intensive PFMT with standing exercises had reduced incontinence, a shorter duration of incontinence duration, and improved quality of life. A Meta-analysis (21) indicates that preoperative PFMT (initiated between four weeks to one day pre-surgery) significantly reduces the risk of urinary incontinence three months post-RP by 36%. The findings also suggest that preoperative PFMT may aid early incontinence recovery, although it may not significantly impact long-term incontinence rates beyond six months. Therefore, it is recommended to start PFMT 3–4 weeks preoperatively to increase neuromuscular adaptation (7) and reserve, advocating for prolonged, higher frequency and/or intensity PFMT before surgery (22).

In addition, all surgeries in this study were performed by the same highly experienced surgeon, which significantly reduced inter-operator variability at the source and achieved a high degree of standardization in operative procedures. On the other hand, it also, to some extent, “leveled out” inter-patient differences and the independent effects of traditional influencing factors (4), resulting in no statistically significant associations observed within this cohort. Specifically: (I) For BMI and prostate volume, both exhibited a narrow distribution and limited effect size within this cohort, and their correlations became non-significant after multivariate adjustment; (II) The urethral reconstruction technique was implemented with high consistency under the single-surgeon framework, markedly reducing intra-technique variability; (III) The number of nerve-sparing cases was limited, and their independent effect further diminished after adjusting for covariates. In addition, the absence of key anatomical parameters such as MUL (23, 24) may have further masked the impact of subtle technical differences on functional outcomes and complication risks.

### 4.1 Limitations

This study had certain limitations. First, the study participants were all from the same tertiary hospital, which means that the findings may be more applicable to patients in hospitals with a higher level of medical care and may not be generalisable to hospitals with average medical conditions. This concentrated source limited data extensibility. Second, this study lacks external validation and the current conclusions are based solely on a single dataset. This restricts the broad applicability of the results, because the lack of validation from different data sources may affect the reliability of the conclusions. Additionally, the study did not include the MUL indicator from the MRI data. Because most patients underwent MRI at referring hospitals with non-standardized scanning protocols, and the MRI reports obtained at our institution did not routinely include the MUL parameter; moreover, most patients underwent surgery within 1–2 days after admission, making it impossible to repeat MRI under a unified standard. This omission could have had some impact on the study results; therefore, future research should address this aspect. In summary, although this study provides some insights, its limitations warrant attention. Future research should consider expanding sample sources, increasing external validation, and supplementing critical variables to enhance the comprehensiveness and applicability of this study.

## 5 Conclusions

The fast-twitch muscle score should be regarded as a potential, functional-level predictor and cannot be considered a direct substitute for MUL. A nomogram was created in this study to better assist clinicians in estimating the risk of postoperative urinary incontinence. This tool visually displays the probability of urinary incontinence for different combinations of age and fast-twitch muscle scores, allowing doctors to assess the risk level for individual patients more accurately. Using a nomogram, doctors can provide patients with clearer preoperative risk assessments, enabling the formulation of personalised treatment and care plans.

### 5.1 Relevance to clinical practice

This study presents significant advancements in understanding and managing urinary incontinence following RP, highlighting the predictive value of age and functional-level of fast-twitch muscle scores. By identifying fast twitch muscle scores as modifiable risk factors through pelvic floor muscle training, this study offers a promising avenue for improving postoperative outcomes. This underscores the necessity for integrating patient-specific factors into preoperative evaluations, thereby promoting a shift towards more personalised healthcare strategies.

These findings advocate the integration of a tailored approach that can significantly enhance patient counselling and decision-making processes. By considering individual parameters, healthcare providers can develop more effective management strategies that cater to the specific needs of patients at risk of postoperative urinary incontinence, potentially leading to better patient experiences and outcomes. A notable advancement introduced by this study is the establishment of a practical nomogram tool designed to facilitate precise preoperative evaluation. This tool allows clinicians to visualise and estimate the probability of urinary incontinence based on a range of patient-specific data, thereby enabling more accurate treatment planning and realistic recovery expectations. In addition to the nomogram, this study emphasised the indispensable role of specialised incontinence care nurses. These practitioners play a crucial role in conducting accurate assessments and guiding patients through targeted pelvic floor muscle exercises, which are crucial for the effective recovery and long-term management of urinary incontinence. Their expertise ensures that patients receive the necessary support and training, thereby enhancing the overall recovery process.

## Data Availability

The original contributions presented in the study are included in the article/Supplementary Material. Further inquiries can be directed to the corresponding authors.
